# Role of bZIP Transcription Factors in Plant Salt Stress

**DOI:** 10.3390/ijms24097893

**Published:** 2023-04-26

**Authors:** Haotian Liu, Xun Tang, Ning Zhang, Shigui Li, Huaijun Si

**Affiliations:** 1 College of Life Science and Technology, Gansu Agricultural University, Lanzhou 730070, China; 2 State Key Laboratory of Aridland Crop Science, Gansu Agricultural University, Lanzhou 730070, China

**Keywords:** salt stress, bZIP transcription factors, molecular regulation, growing development

## Abstract

Soil salinity has become an increasingly serious problem worldwide, greatly limiting crop development and yield, and posing a major challenge to plant breeding. Basic leucine zipper (bZIP) transcription factors are the most widely distributed and conserved transcription factors and are the main regulators controlling various plant response processes against external stimuli. The bZIP protein contains two domains: a highly conserved, DNA-binding alkaline region, and a diverse leucine zipper, which is one of the largest transcription factor families in plants. Plant bZIP is involved in many biological processes, such as flower development, seed maturation, dormancy, and senescence, and plays an important role in abiotic stresses such as salt damage, drought, cold damage, osmotic stress, mechanical damage, and ABA signal response. In addition, bZIP is involved in the regulation of plant response to biological stresses such as insect pests and pathogen infection through salicylic acid, jasmonic acid, and ABA signal transduction pathways. This review summarizes and discusses the structural characteristics and functional characterization of the bZIP transcription factor group, the bZIP transcription factor complex and its molecular regulation mechanisms related to salt stress resistance, and the regulation of transcription factors in plant salt stress resistance. This review provides a theoretical basis and research ideas for further exploration of the salt stress-related functions of bZIP transcription factors. It also provides a theoretical basis for crop genetic improvement and green production in agriculture.

## 1. Introduction

Land is an essential resource for human social production activities and sustainable development. However, population growth and massive greenhouse gas emissions are leading to rising global temperatures and changes in the world’s climate, further exacerbating soil salinization, which is detrimental to plant growth and development [1,2]. Salinized soil leads to a decrease in seed germination rates and inhibits crop growth, thereby constraining agricultural development and threatening food security. Soil salinization is one of the main adverse environmental factors affecting agricultural production and food security [3]. In order to cope with environmental change, plants have evolved complex signaling pathways, which are usually composed of receptors, secondary signals, plant hormones, and signal transducers [4]. Transcription factors (TFs), also known as trans-acting factors, are important components of abiotic stress signal transduction pathways. Transcription factors are involved in the process of plant resistance by binding to different *cis*-elements and regulating the expression of a family of related genes. Typical transcription factors generally consist of four parts, namely, the DNA-binding domain, transcriptional regulation domain, nuclear localization signal (NLS), and oligomerization site. The combined action of these structural domains determines the time, space, and mode of action of the regulatory functions of transcription factors [5]. Many transcription factors act as major regulators to select genes, controlling the determination of cell type, development pattern, and specific pathway control [6]. When plants are exposed to biotic and abiotic stresses, such as salinity, drought, low temperature, high temperature, and diseases, they produce and transmit a series of signals to stimulate transcription factors. Subsequently, transcription factors combine with corresponding *cis*-acting elements to activate RNA polymerase and transcribe complexes, thereby initiating the transcription and expression of specific genes. Finally, gene products begin to respond to the signal [7]. As the basic role of transcription factors involves improving plant stress resistance, the functional study of these genes is also crucial in plant stress-resistance biotechnology engineering. Basic leucine zipper (bZIP) transcription factors, the most widely distributed and conserved proteins in terms of eukaryotic transcription factors, have important regulatory functions in many biotic and abiotic stresses [8]. BZIP transcription factors are characterized by a basic region and a leucine zipper domain. The sequence of the basic region is relatively conserved and consists of about 20 amino acid residues. The fixed nuclear localization structure N-(X) 7-R/K can specifically bind to DNA *cis*-elements. The leucine zipper region is not conserved, consisting of one or more repetitive regions and containing a large number of hydrophobic residues via oligomerization [8]. Transcription factors can be divided into several categories according to the characteristics of DNA binding regions. Among them, there are four main categories related to plant stress resistance: bZIP [9], WRKY [10], AP2/ERF [11], and MYB [12]. As one of the most widely distributed and function-diverse transcription factor groups, bZIP transcription factors play an important regulatory role in the plant stress response [13]. Plants inevitably face a variety of stresses, including salinity, drought, waterlogging, and cold damage, during their growth and development. Plant bZIP transcription factors play an extremely important role in resisting these adverse natural environments. bZIP transcription factors are induced by abiotic environmental stresses, including salinity, drought, cold damage, and heavy metals. BZIP has been found to confer salt resistance in *Arabidopsis* [14], *Lycopersicon esculentum* Mill [15], *Helianthus annuus* [16], *Oryza sativa* [17], and other plants [18].

According to the Chinese Ministry of Agriculture statistics, less than 10% of the world’s land is suitable for cultivation, and most of the land is in an adverse state, e.g., saline or alkaline land. With the deterioration of the environment and the growing population, it is necessary to cultivate economic crops that can grow under various stresses. Transcription factors have the potential to enhance the comprehensive resistance of plants. This paper briefly introduces the structural and functional characteristics of bZIP transcription factors and focuses on their role in plant salt stress resistance and molecular mechanisms.

## 2. Structural Characteristics of bZIP Transcription Factors

**Figure 1:**
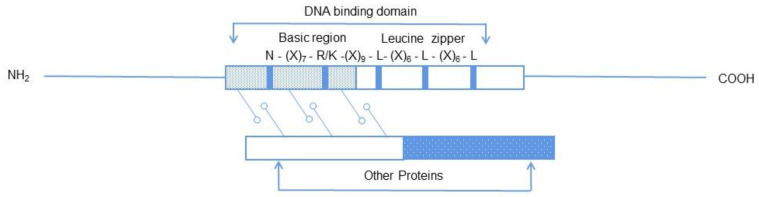
Structure diagram of bZIP transcription factor.

BZIP is one of the most studied transcription factor gene groups. It has been sequenced in many plants, and studies have found that the factors of the group are different in the corresponding species (Table 1) [19]. Each part of the bZIP transcription factor genome contains a highly conserved structure with a length range of 60–80 amino acids, consisting of a highly conserved DNA-binding alkaline region and a variable leucine zipper region. The sequence of the basic region is relatively conservative, consisting of about 20 amino acid residues, and contains a fixed nuclear localization structure, i.e., N-(X) 7-R/K, that can specifically bind to DNA *cis*-elements near its N-terminal. The leucine zipper region is not conserved and contains two α-helix, and each sixth amino acid residue contains one leucine (Leu) or another hydrophobic isoleucine, valine, methionine, etc. (Figure 1) [7]. The N-terminus of the leucine zipper region can be associated with the acidic domain to form homologous or heterologous dimers, which then realize transcriptional inhibition and activation. Before bZIP transcription factors bind to DNA, leucine can cause two α-helix dimerization to form homologous or heterologous dimers, directly bind to DNA in the basic region of the N-terminus of the peptide chain in the form of dimers, and then realize transcriptional inhibition and activation [9]. 

The light part is alkaline leucine, the dark blue part is a highly conserved residue, and the superscript is the corresponding sequence. The N-terminus of the transcription factor contains an acidic activation region that binds to DNA in the form of a dimer, and the base region at the N-terminus of the peptide chain directly binds to DNA. Leucine is sometimes replaced by isoleucine, valine, etc.

bZIP transcription factors in plants are currently divided into 10 subgroups according to their structures: A, B, C, D, E, F, G, H, I, and S [9]. The bZIP transcription factors of different subgroups perform unique functions in different species. bZIP transcription factor A genes can participate in the activation of the ABA signal, regulate the expression of downstream stress-resistance genes, and greatly improve the drought resistance of plants. For example, *AtbZIP12* and *AtbZIP39* can regulate gene expression by competing for the same binding sites within the *AtEm1* gene promoter during plant development [9,30]. bZIP transcription factor B genes are mainly involved in the regulation of the salt stress response and endoplasmic reticulum stress response, such as *AtbZIP17* and *AtbZIP28* in *Arabidopsis thaliana* [31]. D subgroup genes are involved in two different processes: defense against disease and physiological growth. D proteins may be involved in different signal transduction pathways at the PR promoter level in response to bacterial infection [32]. Three conserved proline-rich regions at the N-terminus of histone G have the potential for transcriptional activation and may regulate the expression of storage protein genes, suggesting that histone G may also affect seed maturation [33].

## 3. Effects of Salt Stress on Plants

Soil salinization has become an increasingly serious worldwide problem, significantly affecting the growth and yield of crops and posing a major challenge to plant breeding [34].

When plants grow in highly saline soil, the first problem is that the original water balance is disturbed. The damage to plants caused by salt stress is called salt damage, including primary salt damage and secondary salt damage. Primary salt damage refers to the direct effect of salt ions, causing major damage to the cell membrane. Secondary salt damage refers to the indirect effect of salt ions, which is manifested as osmotic stress and makes it difficult for plant roots to absorb water, resulting in water and nutrition deficits. With the increase in salt concentration in the soil, the concentration of the soil solution will also increase, resulting in an increase in osmotic pressure, which weakens the absorption of water by plants [3]. When the salt concentration in the soil is too high to cause the osmotic pressure of the soil solution to be higher than the osmotic pressure of the plant cells, the plant cannot absorb water from the soil and can even dehydrate plant roots, resulting in physiological drought or even the death of the plant [35].

On the one hand, salt stress directly affects the cell membrane lipid bilayer arrangement and membrane protein structure so that lipid membrane permeability increases and leads to membrane lipid peroxidation, thus affecting the normal physiological function of the membrane [36]. When plants are under salt stress, cells continue to lose water, causing changes in cell turgor and changes in osmotic potential. When the cell membrane and plasma membrane of plants are destroyed, the selective permeability of the cell membrane is reduced or even lost, so that a large number of beneficial ions such as Ca^2+^ and K^2+^ in the cell leak out; harmful ions such as Na^+^ and Cl^−^ accumulate in the cell, and the ion balance in the cell is destroyed. This also results in damage to the cell membrane, organelle membrane, and organelle structure; decreases the function, chlorophyll content, and photosynthetic rate; and increases ribonuclease activity, etc. [37]. As a result, the plant’s resistance is weakened by stress damage [38].

On the other hand, under salt stress, plants accumulate more reactive oxygen species (ROS); if these ROS cannot be promptly removed, they will cause oxidative stress. Excessive accumulation of ROS can destroy the structure of cells; damage DNA, lipids, proteins, and other biological macromolecules; and eventually lead to cell death [39]. ROS has strong oxidizing power; it has a destructive effect on unsaturated lipids, proteins, nucleic acids, and other biological molecules, causing enzyme inactivation, pigment decolorization, protein degradation, and lipid peroxidation [40]. ROS is a by-product of aerobic metabolism in plants, so it is continuously produced in plants. Common active oxygen substances include hydrogen peroxide, superoxide, hydroxyl radicals, and singlet oxygen [41]. Under normal conditions, ROS are produced in plants and are continuously eliminated. These two processes form a dynamic balance, thereby maintaining reactive oxygen species at a relatively constant low level [42]. At this level, ROS are necessary messengers in the redox signaling pathway, which can initiate and participate in the signaling network to regulate physiological processes such as growth, development, defense response, and adaptability to biotic or abiotic stress [43,44,45,46]. However, both biotic and abiotic stresses can induce an increase in ROS production and cause them to reach harmful levels, resulting in oxidative damage to the cell structure and intracellular substances such as the cell membrane, protein, and DNA [47].

## 4. Plant bZIP Transcription Factors Interact with Genes in Response to Salt Stress

**Figure 2:**
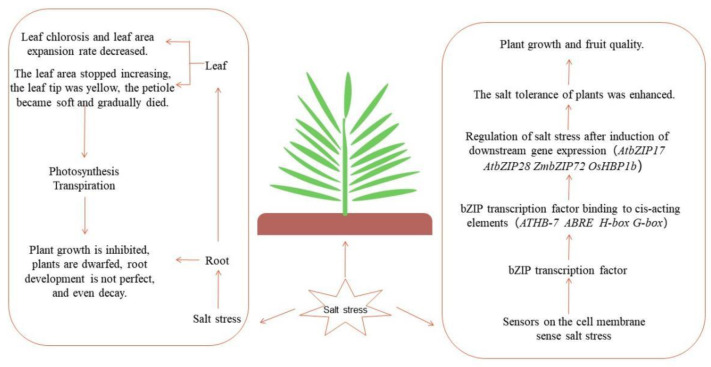
Gene network controlling plant salt tolerance.

Plants can face adverse conditions such as salinity, drought, heavy metals, and extreme temperatures during growth. In order to adapt to abiotic stress conditions, plants have evolved defense mechanisms through stress-induced genes, such as phosphatases, protein kinases, and transcription factors. The bZIP transcription-factor-mediated abiotic stress response network is an important mechanism of plant response to various abiotic stresses. In the process of exploring plant adaptation to the environment, it was found that bZIP transcription factors in plants can interact with ABRE(CCACGTGG) elements [48], PB(TGAAAA) elements, GLM (GTGAGTCAT) elements [49], G-box (CACGTG) elements [50], and H-box (CCTACC) and ACE (ACGT) elements (Figure 2), to induce the expression of downstream genes.

When plants are under salt stress, in addition to regulating the intracellular K+/Na+ concentration ratio, in order to adapt to the new environment, these plants constantly adjust their growth, and the regulation process requires a variety of transcription factors regulating downstream gene activity to be involved. The expression of plant bZIP transcription factors is mainly induced by plant hormones such as salicylic acid (SA), ethylene (ET), gibberellin (GA), methyl jasmonate/jasmonic acid (MeJA), and abscisic acid (ABA) [51]. ABA is mainly involved in various abiotic stresses, such as drought or osmotic-induced stomatal closure, and induced tolerance to water, salt, hypoxia, and low-temperature stress [52]. SA is an important signal for the molecular mechanism of plant response to pathogenic biotic stresses. When plants are infected by pathogens, the initiation of SA-mediated signaling pathways involved in disease-resistance regulation helps these mechanisms to improve stress tolerance and productivity [53]. JA and ET are usually associated with defense against necrotic pathogens, insects, and herbivores [54]. Under these salt stress conditions, elevated levels of the plant hormone abscisic acid (ABA) in plant tissues trigger the adaptation of plants, which is essential for their survival and productivity. Therefore, the mechanism of the ABA-induced expression of related genes is critical for the plant response to salt stress [55,56]. The ABA response element (ABRE) with an ACGT response core sequence is a well-known *cis*-element found in the promoters of genes involved in plant stress response and ABA-induced expression [57]. BZIP transcription factors can regulate plants under salt stress by binding to ABRE, making plants resistant. Therefore, the bZIP transcription factor is also named ABRE binding protein or ABRE binding factor. ABRE response element and AREB protein are extremely important for the response and regulation of abiotic stress in plants under high salt conditions [58]. High salt levels can induce the expression of the *bZIP* gene. The bZIP transcription factor binds to the ABA response element (ABRE) and activates many resistance-related genes [9]. The bZIP transcription factor regulates the expression of these genes by interacting with specific *cis*-regulatory sequences in the promoter region of these stress-responsive genes, thereby enhancing stress tolerance. Regulation of bZIP expression levels and patterns, as well as changes in post-translational protein activity, often help activate various signaling pathways [9].

Because salt stress affects the absorption of water in the roots, the leaves lose green, the leaf area expansion rate decreases, the leaf area stops increasing, the leaf tip becomes yellow, the petiole becomes soft, and the leaves gradually die. This affects photosynthesis and transpiration, resulting in decreased plant quality and reduced yield. Salt stress can cause changes in the internal environment of plants; the original metabolic balance is broken, resulting in a large accumulation of harmful substances, and cell membrane structure is destroyed, causing metabolic disorders in the body. In order to maintain the environmental balance in the body, plants will initiate their own defenses to cope with salt stress. bZIP transcription factor-mediated salt stress response network is one of the important mechanisms for plants to cope with adverse environmental conditions. bZIP transcription factors in plants can interact with ABRE (CCACGTGG) elements, PB (TGAAAA) elements, GLM (GTGAGTCAT) elements, G-box (CACG-TG) elements, and H-box (CCTACC) and ACEs (ACGT) elements, to induce the expression of downstream genes. The expression of these downstream genes can reduce the content of MDA and H_2_O_2_, increase the activity of CAT, SOD, and POD, and improve the ability of plants to cope with salt stress.

## 5. bZIP Transcription Factors Related to Salt Stress in Plants

**Figure 3:**
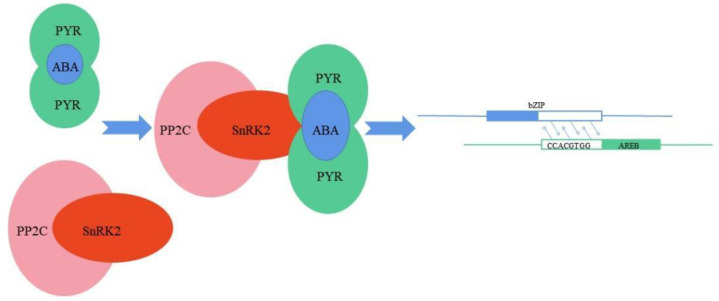
PP2Cs bind to and dephosphorylate SnRK2, blocking ABA signaling pathway and stress response. Salt stress and other stress environments induce the production or release of ABA. ABA binds to the receptor PYR/PYL/RCAR protein to form a complex, and binds to and inhibits PP2C. SnRK2 is released from the PP2C-inhibited state, and the activated SnRK2 phosphorylates the downstream bZIP transcription factor and up-regulates its transcription level after bZIP binds to AREB. ABA induces the expression of bZIP transcription factors through this pathway to respond to salt stress, and activate ABA signaling pathway and stress response process.

A large number of bZIP transcription factors are associated with salt stress in plants (Table 2). In the model plant *Arabidopsis thaliana*, 75 bZIP transcription factors were found [9]. In *Arabidopsis*, the alkaline leucine zipper transcription factor was found to be involved in the ABA-dependent signal transduction pathway under high-salinity conditions [59]. Two ABRE binding proteins, AREB1 and AREB2 (ABA response element), encoding basic leucine zipper (bZIP), were screened using the Yeast One-Hybrid system [58]. AREB1 and AREB2 belong to the ABREs that can act on the expression of genes involved in the salt stress response. The transcription of AREB1 and AREB2 genes was up-regulated after *Arabidopsis thaliana* was treated with sodium chloride. In a transient reverse-transcription experiment using *Arabidopsis* leaf protoplasts, both AREB1 and AREB2 proteins activated the transcription of a reporter gene driven by ABRE, and it was found that these factors can activate the ABA response promoter in protoplasts (Figure 3) [48]. In addition, published *Arabidopsis* transcriptome data showed that the expression of the *AtbZIP1* transcription factor in roots was induced by salt stress [60]. Through phenotypic experiments on *AtbZIP1* T-DNA insertion mutant *Arabidopsis* plants after ABA treatment, it was verified that *AtbZIP1* is involved in ABA-dependent signaling pathways. *AtbZIP1* can regulate the sensitivity of plants to ABA treatment and the expression of downstream ABA response genes by binding to the ABRE element, thereby participating in the ABA signaling pathway of plants [61]. The above results indicate that ABRE binding proteins can specifically bind to bZIP transcription factors and participate in ABA-dependent signal transduction to resist salt stress. Liu et al. found that *Arabidopsis thaliana* homologs *AtbZIP17* and *AtbZIP28*, which share similar functions with ATF6 (a type 2 membrane-bound transcription factor), can interact with the molecular chaperone binding protein BiP/Grp78 in the endoplasmic reticulum (ER) to induce salt stress-related gene expression [62]. The endoplasmic reticulum is an important organelle for synthesizing cellular proteins, essential lipids, and complex proteins in organisms. Peptides synthesized by ribosomes enter the ER with the assistance of molecular chaperones, then fold, process, add glycosyl, etc., and finally form proteins with specific functions. Protein folding in the endoplasmic reticulum produces misfolding [63]. Misfolded proteins are either eventually further folded with the assistance of molecular chaperones or enter the ER-associated degradation (ERAD) process to be degraded [64]. The organism achieves homeostasis through this process and maintains normal growth and development. Abiotic stress up-regulates the expression of molecular folding enzymes, enhances the folding ability of endoplasmic reticulum protein, leads to transient attenuation of translation, and activates ER quality control (ERQC) [65]. ATF6 is an ER membrane stress-conduction protein; its N-terminal facing the cytoplasm has a bZIP structure, its C-terminal is a large ER cavity domain, and it can interact with ER molecular chaperone binding protein BiP/Grp78 [66]. In normal cells, BiP binds to the cavity-side domain of *bZIP17/28*, thereby retaining *bZIP17/28* in the ER. Under salt stress, BiP is surrounded by a large number of unfolded proteins, releasing *bZIP17/28*, resulting in *bZIP17/28* leaving the ER [67,68]. Then, *AtbZIP17* and *AtbZIP28* are transported to the Golgi apparatus and cleaved by two subtilisin-like serine proteases, i.e., S1P and S2P. The cleaved N-terminal domain forms a functional bZIP transcription factor, which enters the nucleus and induces stress-related gene expression [69].

In a study of soybean, it was found that the overexpression of genes *GmbZIP44*, *GmbZIP62*, and *GmbZIP78* could significantly enhance soybean salt tolerance compared with wild-type plants when soybean plants were in the seedling and bolting stage. The salt tolerance of these genes was positively correlated with the proline level in transgenic plants. GLM (GTGAGTCAT), ABRE (CCACGTGG), and ABRE (CCACGTGG) were used in a Yeast One-Hybrid assay and an in vitro gel transfer assay. The study also showed that *GmbZIP44*, *GmbZIP62*, and *GmbZIP78* are negative regulators of ABA signaling, and may be involved in ABA signaling by up-regulating ABI1 and ABI2, playing a role in salt stress tolerance by regulating a variety of stress response genes [70]. Compared with wild-type (WT) *Arabidopsis* plants, transgenic plants overexpressing *GmbZIP132* showed reduced abscisic acid sensitivity and an increased water loss rate. At the germination stage, transgenic plants were more tolerant to salt treatment than wild-type plants. In a Yeast One-Hybrid assay, *GmbZIP132* was able to bind to the GCN4-like motif (GLM) (5′-GTGAGTCAT-3′). The gene expression of *GmbZIP132* was up-regulated under high-salt treatment. Among all the organs analyzed, *GmbZIP132* had the highest expression in cotyledons and stems. In terms of the plant stress-response element ABRE, these results indicate that the bZIP transcription factor in soybean is a response to salt stress-related genes, and its overexpression can improve the salt tolerance of transgenic plants [71].

Studies have found that 89 rice bZIP transcription factors are widely involved in abiotic stress response [48]. *OsbZIP23* is a member of the bZIP transcription factor family in rice. Transgenic rice overexpressing *OsbZIP23* showed significantly increased tolerance to drought and high-salt stress and sensitivity to ABA. The results showed that *OsbZIP23*, as a transcriptional regulator, can respond to abiotic stress by regulating the expression of a wide range of stress-related genes through ABA-dependent regulatory pathways. Therefore, it is believed that *OsbZIP23* has ABA-dependent drought and salt stress tolerance and has high potential application value in the genetic improvement of salt stress resistance [72]. ABA-induced bZIP transcription factor genes *OsbZIP05* and *OsbZIP66* were up-regulated under dehydration and salt stress conditions, indicating that these genes are involved in the ABA-dependent stress signal transduction pathway. In addition, other genes can be induced by the expression of bZIP transcription factors to the corresponding stress [72]. For example, overexpression of the NAC gene also helps to improve the salt tolerance of rice. By analyzing the transcriptome data of overexpressing plants, it was found that *OsNAC106* could induce the expression of *OsbZIP23* [73]; *OsNAC022* can also target the stress-response genes of *OsbZIP23* and other transcription factors [74].

In an in-depth investigation of the regulation of stress-responsive genes in tomato plants, a bZIP-like AREB was identified from tomato cDNA and named *SlAREB*. In tomatoes, the AtRD29a promoter and the SlLAP (leucine aminopeptidase) promoter can drive the expression of the *SlAREB* transiently trans-activated luciferase reporter gene under exogenous salt stress, indicating that ABA-dependent post-translation can activate the SlAREB protein. An electrophoretic mobility shift assay also demonstrated that the recombinant DNA binding domain SlAREB protein could bind to the AtRD29a promoter and SlLAP promoter regions. SlAREB protein increased both tolerances to water deficits and high salt stress as well as the water content in *Arabidopsis* and tomato plants. These results indicate that the function of *SlAREB* in tomatoes is to regulate salt stress response genes, and its overproduction improves plant tolerance to water deficits and salt stress [75,76].

A total of 65 potato bZIP genes were identified in a study of potatoes. According to the potato bZIP phylogenetic tree, these 65 genes were divided into 13 groups. *StbZIP65* was heterologously transformed into *Arabidopsis thaliana*. After salt stress treatment, it was found that overexpression lines exhibited enhanced salt tolerance. No growth difference was observed between wild-type and transgenic *Arabidopsis* under normal conditions. However, under salt stress, the main root length of transgenic *Arabidopsis* was longer than that of wild-type plants, and transgenic *Arabidopsis* grew stronger than wild-type plants. The StbZIP65-GFP fusion protein was observed in tobacco epidermal cells via a subcellular localization assay, revealing that *StbZIP65* was expressed in the nucleus [77]. It was found that some *StbZIP* genes were induced by different stress conditions. The expression of *StbZIP25*, a homolog of *AtbZIP36*/*ABF2*, was significantly up-regulated under salt stress. The StbZIP25 protein is located in the nucleus and is heterologously transformed into *Arabidopsis thaliana*. Overexpression of *StbZIP25* enhances the salt tolerance of *Arabidopsis thaliana* [78].

In a study of the heterologous transformation of pepper gene *CabZIP1* into *Arabidopsis thaliana*, the salt tolerance and drought resistance of transgenic plants were also improved. Chromatin immunoprecipitation showed that *CabZIP1* bound to the G-box element in the *CaPR-1* gene promoter. Taken together, these results suggest that *CabZIP1* functions as a transcriptional regulator of the *CaPR-1* gene to improve plant tolerance to abiotic stresses such as salt stress [79].

*ZmbZIP72* is a bZIP transcription factor cloned from maize, which was found to improve the drought- and partial salt-tolerance of transgenic *Arabidopsis* plants via heterologous transformation. In addition, overexpression of *ZmbZIP72* enhanced the expression of ABA-induced genes such as RD29B, RAB18, and HIS1-3. These results suggest that the ZmbZIP72 protein functions as an ABA-dependent transcription factor in positively regulating abiotic stress tolerance [80].

Through transgenic plants with the *bZIP* gene, it was found that transgenic plants could significantly improve the stress resistance of plants. The difference is that *bZIP* can improve stress resistance by regulating different downstream genes. During the study of bzip transcription factors in *Jatropha*, 50 *JcbZIP* genes were identified and divided into 10 groups. The results of gene expression patterns and real-time quantitative PCR showed that *JcbZIP34*, *JcbZIP36*, *JcbZIP49,* and *JcbZIP50* were key resistance-related genes under drought and salt stress conditions. According to the results of *cis*-elements and phylogenetic analysis, *JcbZIP49* and *JcbZIP50* may be involved in the response to drought and salt stress, while *JcbZIP34* and *JcbZIP36* may also play an important role in seed development and response to abiotic stress [81].

Through transgenic plants with the bZIP gene, it was found that transgenic plants could significantly improve the stress resistance of plants. The difference is that bZIP can improve stress resistance by regulating different downstream genes. MiRNA plays an important role in the regulation of crop response to abiotic stress. By analyzing the transcription factor binding sites (TFBS) motifs in the 800 bp upstream sequence of the TSS of 96 miRNAs, 30,461 potential TFBS were found, belonging to 38 TF families, including bZIP transcription factors. Through the regulation of target genes, they stimulate signal transduction substances, stress-protective substances, secondary metabolites, etc., to respond to adversity stress, thereby improving plant tolerance to adversity stress. The regulation of miRNA on bZIP transcription factors in salt stress remains to be further studied [82]. Function analysis of lncRNAs showed that they were associated with salt stress response-related genes bZIP, MYB, and WRKY. Subsequent studies on the comprehensive function of lncRNA will help to deepen the molecular mechanism of lncRNA regulating bZIP transcription factors to improve plant salt tolerance [83].

**Table 2:** A series of bZIP transcription factors are involved in the regulation of various plant salt stress.

Species	bZIPs	References
*Arabidopsis thaliana*	*AtbZIP1*	[61]
	*AtbZIP17*	[31]
	*AtbZIP18*	
*Oryza sativa*	*OsbZIP23*	[73]
	*OsbZIP05*	[84]
	*OsABF1*	[85]
	*OsABF2*	[57]
*Glycine max*	*GmbZIP44*	[70]
	*GmbZIP62*	
	*GmbZIP78*	
	*GmbZIP1*	[86]
	*GmbZIP132*	[71]
*Jatropha curcas*	*JcbZIP34*	[81]
	*JcbZIP36*	
	*JcbZIP49*	
	*JcbZIP50*	
*Solanum tuberosum*	*StbZIP25*	[77]
	*StbZIP65*	[78]
*Triticum aestivum*	*TabZIP15*	[87]
*Capsicum annuum*	*CAbZIP1*	[79]
*Setaria italica*	*SibZIP35*	[88]
	*SibZIP73*	
	*SibZIP64*	
	*SibZIP49*	
	*SibZIP50*	
	*SibZIP39*	
	*SibZIP38*	
	*SibZIP24*	
*Tamarix hispida*	*ThbZIP1*	[89]

## 6. Functional Characterization of bZIP Gene through Gene Editing

In the process of identifying the function of the bZIP gene through gene editing, it was found that the mode of action and types of bZIP transcription factors were different in different species. The bZIP transcription factors related to stress resistance in *Arabidopsis* are mainly distributed in the A, B, and S subfamilies. *AtbZIP17* is a bZIP transcription factor that encodes a member of the *Arabidopsis* B family. The expression level of *AtbZIP17* is increased under salt stress. The gene is connected to the endoplasmic reticulum membrane through its own C-terminus. Under salt stress, the N-terminus of the gene is released from the endoplasmic reticulum membrane by proteolysis and enters the nucleus to exert its transcriptional activation to improve salt resistance and induce the expression of downstream stress response genes such as *AtRD29A* and *AtRD20* [31]. The *Arabidopsis* S family is also a large family involved in abiotic stress. For example, *AtbZIP11* and *AtbZIP53* mainly respond to salt stress. The *AtbZIP1* gene is induced by salt, drought, and cold. Increased expression and overexpression of the gene were found to significantly reduce the effects of salt and drought on plants. However, *AtbZIP1* is different from *AtbZIP17* in regulating salt stress. *AtbZIP1* regulates the sensitivity of plants to ABA treatment and the expression of downstream ABA response genes by binding to the ABRE element, thereby participating in the ABA signaling pathway of plants to enhance plant stress resistance [61]. The rice bZIP transcription factor genes *OsABI5* and *OsbZIP71* both bind to G-box *cis*-acting elements and interact with bZIP transcription factor C subfamily members to form homodimers or heterodimers. *OsABI5* was cloned from rice panicles. Amino acid sequence alignment showed that it had high homology with *AtABI5* and *HvABI5*-like genes, indicating its functional similarity. The expression of the gene at the transcriptional level responds to ABA and high salt, while the expression level is reduced under low temperatures and drought stress. Overexpression of this gene can increase the sensitivity of transgenic rice to salt, and knockout of this gene can improve the stress resistance of transgenic rice, but reduce the seed-setting rate of seeds [90]. Similarly, the expression of the *OsbZIP71* gene increased under ABA and drought stress while decreasing under salt stress. The transgenic rice overexpressing *OsbZIP71* had significantly improved drought and salt resistance, while the transgenic plants were more sensitive to drought, salt, and ABA after knocking out the gene by RNAi technology [91]. Liao found that soybean *GmbZIP44*, *GmbZIP62,* and *GmbZIP78* belong to subgroup A members, which can regulate the expression of *ABI1* and *ABI2* genes and play an active regulatory role by initiating multiple resistance reactions to improve plant resistance to saline–alkali land and low-temperature environments [87,92].

## 7. Discussion

Soil salinization is the main abiotic stress in crop production worldwide, seriously limiting seed germination, plant growth, and crop production, and endangering food security. About one-third of the world’s land is saline. The problem of soil salinization is particularly prominent in China, and is one of the main obstacles restricting the improvement in agricultural productivity. At present, the area of saline–alkali land in China covers more than 37.5 million mu, and it is increasing every year. For field crops, soil is the substrate for seed germination, and is also the main factor affecting the development, growth, and final yield of plants. The physiological and metabolic characteristics of crops are greatly affected by the environment, but there are relatively few reports on this aspect [93]. Therefore, the mechanism of plant salt tolerance and the cultivation of salt-tolerant crop varieties have become current research hotspots [94].

The function of bZIP transcription factors, especially in plant growth and development, is of great significance for the breeding of high-quality, high-yield, and stress-resistant crop varieties. Increasing numbers of plant transcription factors and their downstream genes have been predicted and verified using bioinformatics and molecular biology methods. Transcription factors are regulated at the post-transcriptional level, especially by directly binding to conserved *cis*-regulatory promoters, so the mechanisms involved in the plant stress response are more complex [95]. Based on this, plant bZIP transcription factors have become promising targets for crop improvement because they can control complex agronomic traits and provide positive regulation for better yield, quality, and stress resistance.

The above may help to prove the importance of bZIP transcription factors in plants. As one of the important reasons for improving plant stress resistance, transcription factors play a vital role in regulating plant processes according to their subunits, with various transcriptional activation functions. Most of the downstream genes regulated by transcription factors are genes encoding proteins or enzymes involved in plant metabolic regulation. Overexpression or inhibition of transcription factors can lead to down-regulation or up-regulation of their downstream target genes, resulting in pleiotropic phenotypes of plants. In order to obtain a correct understanding, it is necessary to study transcription factors and their downstream genes simultaneously. Studies have shown that the response of crops to salt stress and alkali stress is inconsistent. Alkali stress and salt stress usually occur simultaneously, but alkali stress will give plants a high pH environment and inhibit plant growth and development from the roots [96]. Under salt stress, plants will show a physiological response to salt stress through the production and content of internal substances, but this series of physiological responses is regulated by the internal molecular mechanism of the plants [97]. There have been many studies on cucumber, corn, soybean, cotton, tomato, and transcriptome determination under salt stress, and many genes have been found. Previous research revealed that bZIP transcription factors are differentially expressed, and plants with the bZIP gene may significantly increase their salt tolerance. In order to effectively study the salt tolerance of plants, it is now essential to effectively screen relevant genes and have a thorough grasp of the function and mechanism of such genes. A bioinformatics investigation of the transcription factors connected to *bZIP* and implicated in the response to salt stress was carried out. For seeds to germinate normally under salt stress, it was hypothesized that the gene was involved in the regulation of plant hormone signal transduction pathways. In that study, combined with the understanding and research related to bZIP transcription factors on the salt tolerance mechanism of plants, it was shown that transgenic crops with the bZIP transcription factors improved the salt tolerance of crops compared with wild-type plants. Many studies on the mechanism of the bZIP transcription factor have found that it can be induced by salt stress and participate in the regulation of various stress responses. This gene group can induce the expression of key resistance genes such as ABA, NAC, ZIP, OFB, BZ, and ABI transcription factors by combining with the ABRE element, PB element, GLM element, G-box element, and other action elements to improve the tolerance of plants to corresponding stresses [98,99,100,101,102].

Growing numbers of plant bZIP transcription factors have been identified, and as research into these proteins becomes more in-depth, their classification and distribution will progressively come to light. Additionally, progress will be made in research regarding molecular structure and function, which will advance knowledge of their involvement in the regulation and expression of genes. Increasing the use of bZIP transcription factors in genetic engineering research opens more pathways for the effective and stable expression of exogenous genes in transgenic plants. Therefore, studying the mechanism of plant germination under salt stress is conducive to improving the germination ability and germination potential of seeds from the source, thereby increasing the final yield of crops. It is of great significance to fully study and cultivate transgenic salt-tolerant crop resources, excavate favorable genes, improve crop varieties, increase farmers’ income in crop-producing areas, and maintain the national grain regional balance. Research regarding bZIP transcription factors should include the following three aspects: the bZIP regulation mode is involved in many biological processes of plant growth and development, such as root morphogenesis and the salt tolerance mechanism of crops. However, the specific regulation mechanism is not clear, so it is necessary to clarify the regulation mechanism of bZIP-mediated downstream genes. Studies have shown that a single bZIP transcription factor in plants involved in abiotic stress responses, such as salt stress and other processes, has functional redundancy, so it is important to analyze the complex redundant regulation mechanism of bZIP subunits. The interaction between bZIP transcription factors or the complex formed via the interaction with other proteins regulates the stress response and flowering time and affects the yield and quality of crops. It is necessary to study the interaction proteins, binding sites, downstream target genes, and biological processes involved in bZIP subunits. Analysis of the molecular regulatory network of bZIP transcription factors is of great significance to enable the use of its characteristics for crop genetic improvement and agricultural green production.

## Figures and Tables

**Table 1:** Number of bZIP transcription factors of some sequenced plants.

Species	Number of bZIP Transcription Factor	References
*Arabidopsis*	78	[9]
*Zea mays*	58	[20]
*Solanum lycopersicum*	69	[21]
*Vitis vinifera*	55	[22]
*Oryza sativa*	89	[23]
*Malus domestica*	114	[24]
*Populus simonii*	45	[25]
*Ziziphus jujuba*	45	[26]
*Poplar*	86	[27]
*Hordeum vulgare*	92	[28]
*Arachis duranensis*	50	[29]

## Data Availability

Not applicable.
